# FiloDetect: automatic detection of filopodia from fluorescence microscopy images

**DOI:** 10.1186/1752-0509-7-66

**Published:** 2013-07-23

**Authors:** Sharmin Nilufar, Anne A Morrow, Jonathan M Lee, Theodore J Perkins

**Affiliations:** 1 Ottawa Hospital Research Institute, 501 Smyth Road, Ottawa, Ontario, K1Y 4E9, Canada; 2 Department of Biochemistry, Microbiology and Immunology, University of Ottawa, 451 Smyth Road, Ottawa, Ontario, K1H 8M5, Canada; 3 School of Electrical Engineering and Computer Science, University of Ottawa, Ottawa, Ontario, K1N 6N5, Canada

**Keywords:** Filopodia, Morphology, FiloDetect, Microscopy image

## Abstract

**Background:**

Filopodia are small cellular projections that help cells to move through and sense their environment. Filopodia play crucial roles in processes such as development and wound-healing. Also, increases in filopodia number or size are characteristic of many invasive cancers and are correlated with increased rates of metastasis in mouse experiments. Thus, one possible route to developing anti-metastatic therapies is to target factors that influence the filopodia system. Filopodia can be detected by eye using confocal fluorescence microscopy, and they can be manually annotated in images to quantify filopodia parameters. Although this approach is accurate, it is slow, tedious and not entirely objective. Manual detection is a significant barrier to the discovery and quantification of new factors that influence the filopodia system.

**Results:**

Here, we present FiloDetect, an automated tool for detecting, counting and measuring the length of filopodia in fluorescence microscopy images. The method first segments the cell from the background, using a modified triangle threshold method, and then extracts the filopodia using a series of morphological operations. We verified the accuracy of FiloDetect on Rat2 and B16F1 cell images from three different labs, showing that per-cell filopodia counts and length estimates are highly correlated with the manual annotations. We then used FiloDetect to assess the role of a lipid kinase on filopodia production in breast cancer cells. Experimental results show that PI4KIII *β* expression leads to an increase in filopodia number and length, suggesting that PI4KIII *β* is involved in driving filopodia production.

**Conclusion:**

FiloDetect provides accurate and objective quantification of filopodia in microscopy images, and will enable large scale comparative studies to assess the effects of different genetic and chemical perturbations on filopodia production in different cell types, including cancer cell lines.

## Background

**Figure 1:**
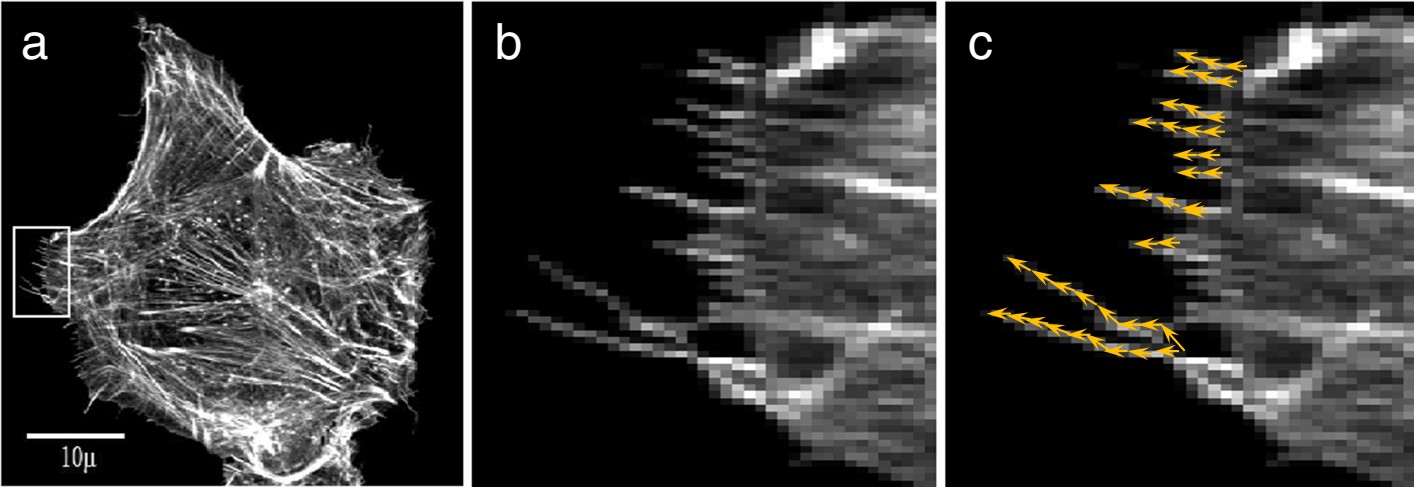
**Images of cells displaying filopodia. (a)** Fluorescence confocal microscopy image of a Rat2 fibroblast cell. **(b)** Close up of the region of subfigure **(a)** shown by the red rectangle. **(c)** Manual labelling of the filopodia are shown by yellow arrows.

Filopodia are thin, finger-like protrusions comprised of tight parallel bundles of filamentous actin (Figure 1(a) and (b)). These protrusions are found at the leading edge of motile cells and are used to sense the cell’s microenvironment [1,2]. Filopodia have been shown to regulate cancer cell motility *in vitro*, and metastasis *in vivo* in mouse experiments [3,4]. As enhanced filopodia production is a characteristic of many invasive cancers, understanding the genetic and chemical factors that regulate filopodia is an important problem. There are presently no algorithms that automatically detect and accurately quantify filopodia in the range of sizes and numbers that are relevant to cancer cell motility. Instead, filopodia are detected by eye, and length or other spatial information are extracted by manually tracing filopodia using image manipulation software. Figure 1(c) shows the manually labeled filopodia. This approach is tedious and slow, limiting the potential size of studies and their statistical power. Our previous work shows that there is high variability in filopodia characteristics, even among genetically identical cells in identical culture conditions [5]. Thus to study filopodia under several different conditions can require tens, if not hundreds, of manually annotated images. High-throughput image-based screens, which may generate thousands or even millions of images, are simply infeasible.

Although there are no automated tools for filopodia detection on cancer cell images, there is considerable work on the closely related problem of tracing neurites in images of neurons. Neurites are any cellular extension of a neuron. Usually, the term refers to axons and dendrites, though it is sometimes used with filopodia. There are sophisticated algorithms for tracing neurites in images, and good public software packages are available [6-12]. The general neurite tracing problem differs in some details from the filopodia detection problem we study. Neurites can have complex branching structures, and it is commonly required to trace them in congested images with multiple cells and many visually crossing neurites. We focus on single-cell images. In these images, filopdia do not branch or cross so extensively as some neurites—although it is not unusual for longer filopodia to cross over other ones, and detection of these filopodia is challenging. Neurites such as axons and dendrites are significantly larger than filopodia, especially in comparison with the cell body. The filopodia we wish to detect can be little more than a pixel wide. Moreover, unless global context is taken into account, other cytoskeletal features within the cell can be confused with filopodia, and the bases of the filopodia, where they enter the cell body, have a considerably heterogeneous appearance.

**Figure 2:**
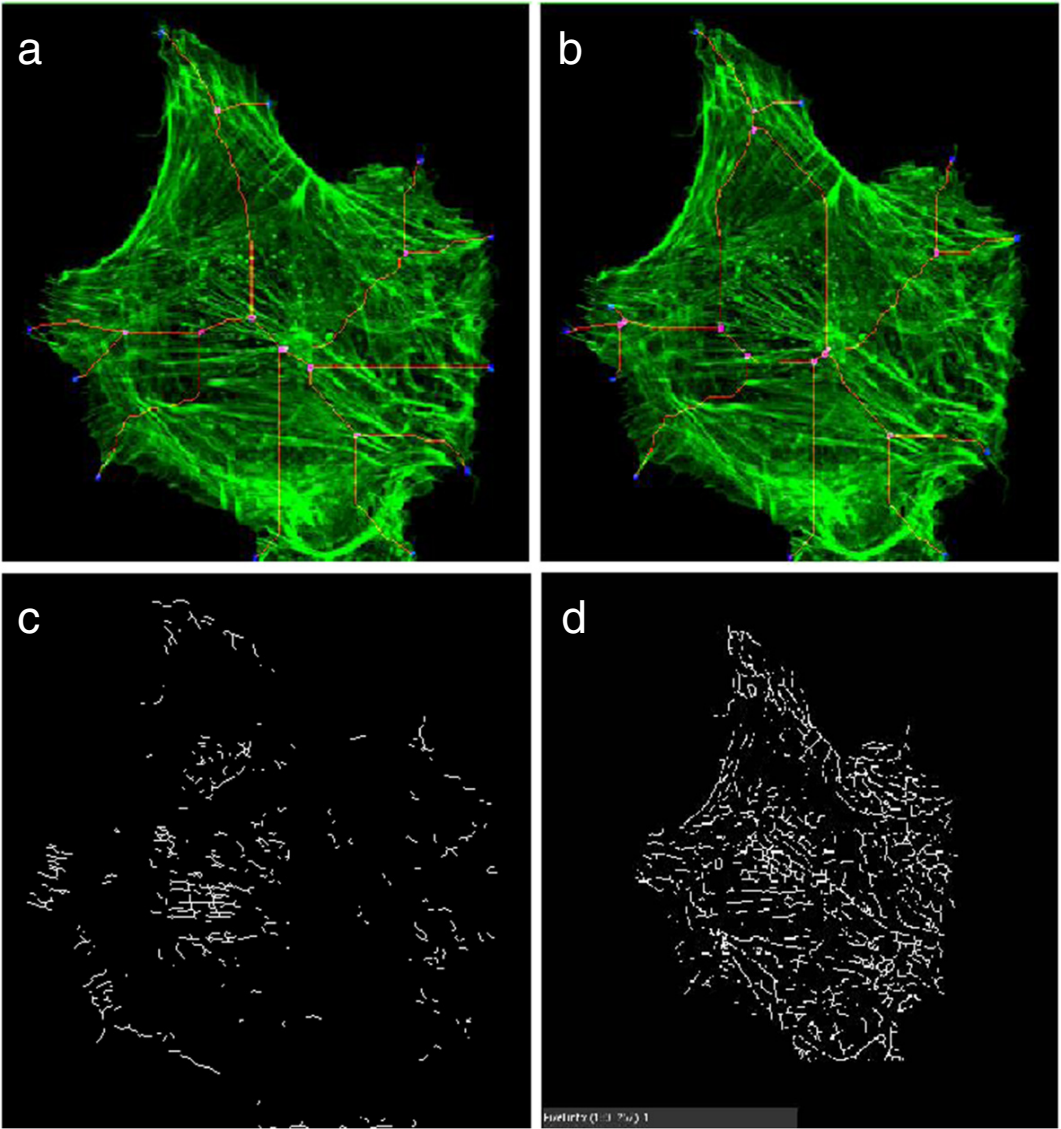
**Filopodia detection in non neural cell of Figure **1. **(a)** result of edge based fTracker (where filopodia tips are represented by blue color and base by pink color) and **(b)** result of intensity based fTracker (where filopodia tips are represented by blue color and base by pink color), **(c)** result of NeuriteQuant (where the white lines represent filopodia) and **(d)** result of WIS-NeuroMath (where the black lines represent filopodia).

The neurite tracing literature includes methods to detect and quantify filopodia on the growth cones of axons during development. However, most of these algorithms are only semi-automated, requiring user interaction to set algorithm parameters for each image or movie. For example [10] target only the larger, and thus easier to detect, filopodia. In a pilot study, we applied three popular software tools, namely fTacker [10], NeuriteQuant [13] and WIS-NeuroMath [11], on our non-neural cell. fTracker, which was originally designed to quantify filopodial dynamics from cultured neurons imaged by time-lapse fluorescence microscopy, works by binarizing the cell image, simplifying the boundary with morphological operators, and skeletonizing. Two types of binarization are used in fTracker: intensity based and edge based. NeuriteQuant is a freely available open-source tool which enables automated morphological analysis of large-scale image data from neuronal cultures or brain sections. Recently proposed software WIS-NeuroMath is based on efficient multiscale detection of edges and fibers in two-dimensional images allowing direct and accurate detection of neurites in various conditions. Figure 2 shows the results of these three existing tools on a sample image. From this figure we can see the existing neurite detection method failed to detect filopodia accurately in non-neural cell. fTracker correctly identifies some filopodia tips, but it traces them back far into the cell, and it misses many other tips. NeuriteQuant and WIS-NeuroMath detect many internal cellular structures, confusing them with cellular protrusions.

Our recent work suggests that filopodia sizes in non-neural cells are lognormally distributed [5]. The few, longest filopodia are not representative of the majority of the filopodia distribution, and thus we must detect all or nearly all filopodia to accurately assess the length distribution. Thus, new algorithms that can accurately detect and quantify filopodia in non-neural cells are greatly needed, as this will allow more rigorous and thorough study of the relationships between filopodia characteristics and the factors that control them.

In this paper, we propose FiloDetect, a fully automated method to detect filopodia from the cell body and measure filopodia length. The approach is inspired by neurite detection methods, including NeuriteQuant and fTracker, but designed in such a way as to avoid the problems they have with our kind of images. We employ intensity-based thresholding and a combination of morphological operations to detect the filopodia. The algorithm is implemented in Matlab and is publicly available at http://www.perkinslab.ca/Software.html. We validated FiloDetect on the non-transformed rodent cell line Rat2 and mouse melanoma cell line B16F1. The Rat2 images used to test the algorithm have been previously manually annotated for filopodia length and number [5], allowing us to assess the accuracy. The B16F1 images were annotated newly for this study.

We then used FiloDetect on a novel dataset, to determine whether expression of the lipid kinase, PI4KIII *β*, impacts filopodia production in breast cancer cells. We were interested in this question because of several lines of evidence implicating a role for PI4KIII *β* in breast cancer and filopodia production: it is activated by eEF1A2 (eukaryotic elongation factor 1 alpha 2) [14], which is amplified in approximately two-thirds of breast tumours [15,16]; it was recently identified as a putative breast cancer driver gene, in a large-scale copy number and gene expression analysis of 2000 breast tumours [17]; and ectopic expression of PI4KIII *β* in fibroblast cells increases filopodia number and length [5,14]. Thus, we hypothesized that PI4KIII *β* may drive filopodia formation in breast cancer cells, potentially enhancing their invasivenes. Our analysis shows this is indeed the case, with PI4KIII *β* involved in both increasing the filopodia length and number in the breast cancer cells.

## Implementation

### Dataset

Experiments were carried out on three datasets from three different cell lines. Rat2 rodent fibroblasts, B16F1 mouse melanoma cells, and BT549 human breast ductal carcinoma cells.

#### *Rat2 dataset*

This dataset consists of a subset of 38 single Rat2, rodent fibroblast, cell images taken from [5]. The details of fixation and imaging of these cells can be found in that publication. In this work, all filopodia at least 0.4 microns long were manually annotated, yielding the total number of filopodia on each cell, as well as the lengths and positions of those filopodia (Figure 1(c)). The subset of Rat2 cells studied in this paper were not genetically altered or chemically stimulated. Out of these 38 cells, 12 images were used in the training phase for the development of the automatic detection method and the remaining 26 images were used to test the method.

#### *B16F1 dataset*

This dataset consists of images of B16F1 mouse melanoma cells, and was used for additional validation of FiloDetect, without any further tuning of parameters. We used five images provided by Dr. J. Schober [18] and seven images provided by Dr. T. Svitkina [19,20]. We call these two groups of images the Schober and Svitkina datasets respectively. We manually annotated these images for filopodia, as described previously [5].

#### *BT549 dataset*

This data set consists of images of BT549, human breast cancer cells, that have been manipulated to express the protein phosphatidylinositol 4-kinase III beta (PI4KIII *β*). The BT549 cells ectopically expressing PI4KIII *β* were generated using the pLXSN retroviral system as described by [21]. Human PI4KIII *β* cDNA was cloned into the pLXSN retroviral expression vector (Clontech). Polyclonal pools of BT549 cells stably expressing PI4KIII *β* were selected with 0.4 mg/ml G418. Cells selected to contain the empty pLXSN vector (EV) were also isolated and used as a control. For filopodia imaging, the cells were seeded onto coverslips in 6-well plates (1×10^5^ cells/well), and allowed to adhere for 24hrs. Cells were then fixed in 3.7% paraformaldehyde, permeabilized with 0.1% Triton X-100, blocked with 1% BSA and stained with Phalloidin-546 (Invitrogen). Following staining, cells were mounted on slides using fluorescence mounting media (Dako). All images were acquired with a 100X NA 1.4 oil immersion objective (Olympus) at 1 airy U on a laser-scanning confocal microscope (IX80, Olympus) with Olympus Fluoview FV1000 software. From each group, empty vector control (EV) and PI4KIII *β* expressing (PI4K *β*), 5 images were used in the training phase to fine-tune parameters and 30 images were used in the testing phase.

### Approach

**Figure 3:**
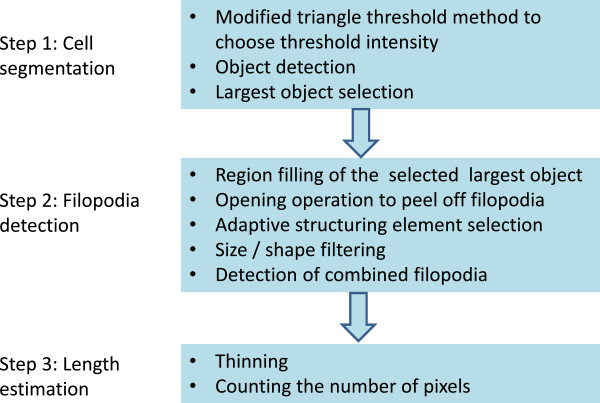
Flowchart showing the steps of FiloDetect system.

As shown in Figure 3, the FiloDetect approach is divided into three basic steps: 1) Intensity thresholding is used to segment the cell body. 2) A series of morphological methods is applied to detect the filopodia. 3) The lengths of the detected filopodia are calculated by thinning them to one pixel wide and counting the pixels that remain. We expand on this outline below.

#### *Step 1: Cell segmentation*

**Figure 4:**
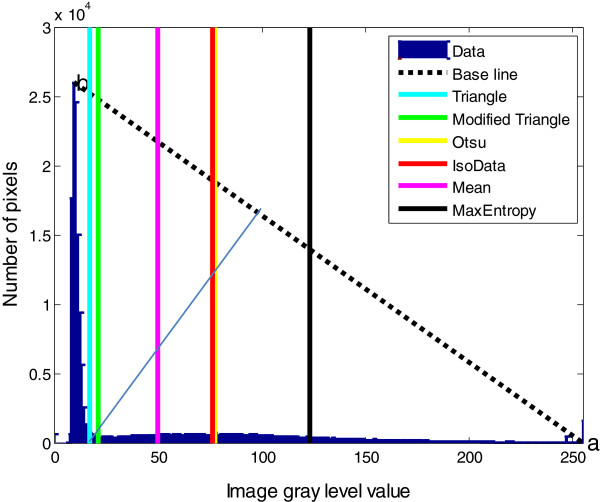
Image pixel intensity histogram with selected threshold values generated by different thresholding methods.

**Intensity thresholding** Intensity thresholding is used to segment an image by setting all pixels whose intensity values are above a threshold to a foreground value and all the remaining pixels to a background value. In broad brush-strokes, it is easy to separate the cell from the background. However, fluorescence confocal microscopy images are usually noisy, and some parts of the cell body typically have low intensity that is very close to the background intensity. Moreover, because we are dealing with fine structures at the periphery of the cell, which may not have high intensity, high precision segmentation is important. Some of our images are background-subtracted and intensity-enhanced for better visibility and some are raw images. As a result, we need an automatic method that can apply for all of these different kinds of images. The popular Otsu’s thresholding method [22,23] which chooses the threshold to minimize the intra-class variance of the black and white pixels, was initially applied by [10] to segment fluorescence microscopy images. However this thresholding technique failed to properly segment the cell body from the background in our images. Here we propose a modified triangle threshold method to segment the cell body from the background. The triangle thresholding method was originally proposed by [24] to segment sister chromatids from microscopy images. In triangle thresholding, a line is constructed between the peak of the histogram *b* to the last non-zero value *a* on the longer tail of the histogram (Figure 4).

**Figure 5:**
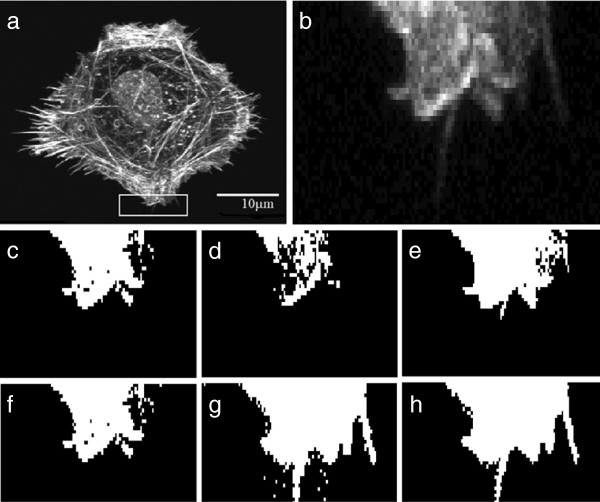
**Results with different thresholding methods. (a)** Original image. **(b)** Magnified part of original image. Thresholded images by: **(c)** Otsu, **(d)** MaxEntropy, **(e)** mean, **(f)** IsoData, **(g)** triangle and **(h)** modified triangle method.

The level where the normal distance between the histogram and the line is maximal is the threshold value (level). However in our case, we searched for local minima at the right side of the threshold value (within 10 neighbouring gray level values). This technique allows us to eliminate some of the background pixels that are detected as foreground pixels in traingle threshold method due to their close intensity level to the foreground pixels. Figure 4 shows the gray level values used in various different popular thresholding methods namely Otsu [22], IsoData [25], mean [26], maximum entropy [27], triangle [24] and the proposed modified triangle methods. The intensity histogram of Figure 4 is generated using the image of Figure 5(a). Figure 5(b) shows an enlargement of part of that image where filopodia can be observed. Figure 5(c–h) show the results of different thresholding methods.

**Cell body selection** There can be substantial noise in images and debris in culture due to cell culturing, fixing and/or imaging conditions. Collectively, these factors result in a variety of objects of different sizes appearing in the thresholded image. Therefore we must select the primary cell from the image. To do this, we use an eight-connected neighborhood to define individual objects. This assigns all ON or white pixels touching vertically, horizontally or diagonally to the same object. The areas of all of the objects present in the image are calculated, and the object with largest area is preserved and considered as the main cell body. All other pixels are set to OFF or zero.

#### *Step 2: Filopodia detection*

After obtaining an initial segmented image, a series of morphological operations is applied to detect the filopodia. Morphology, originally defined as operations on sets, is applied to process images based on shapes [28].

**Figure 6:**
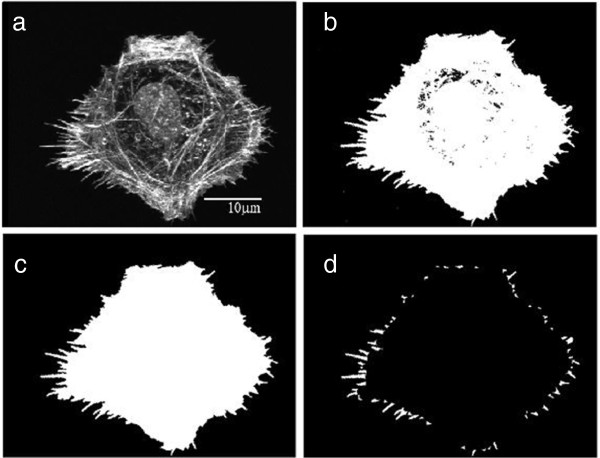
**Step-by-step results for the filopodia detection system. (a)** input image, **(b)** thresholded image, **(c)**image after hole filling and **(d)** final set of split filopodia.

**Region filling** After intensity thresholding, usually some parts of the cell body that have very low image intensity are set to background pixels, as shown is Figure 6(b). The foreground regions can simply be filled by applying a morphological hole filling operation on the thresholded image. A hole is a set of background pixels that cannot be reached by filling in the background from the edge of the image. Figure 6(c) shows the result after the region filling operation.

**Splitting the filopodia from the cell body** To split the filopodia from the main cell body, we begin by applying the morphological opening operation. Opening consists of an erosion step (in which a pixel remains ON only if all pixels in its neighborhood are ON), followed by a dilation step (in which a pixel is turned ON if any pixel in its neighborhood is ON). The opening operation tends to remove small protrusions from the periphery of a larger object. In this case, the fragments removed from the cell body are considered candidate filopodia. However, it is unclear what size of neighborhood is ideal for detecting filopodia. To address this problem, we initially take the neighborhood of a pixel P to be all those pixels whose centers are ≤ 0.5 microns from the center of pixel P. We chose this threshold because the filopodia in our images generally had a width of ≤ 0.4 microns, and thus are eliminated by the opening operation. We further filter objects that are not sufficiently filament-like, by fitting an ellipse to the pixels in the object and discarding objects whose major axis in less than 1.5 times as long as the minor axis. This removes cellular protrusions too thick to be considered single filopodia. We use the remaining objects to get a more precise, cell-specific estimate of filopodia width, by calculating their average minor axis length *L*. We then apply the opening operation again to the original image using a structuring element of radius *L*, generating a revised set of candidate filopodia. Finally, we filter this set to remove objects less than 0.4 microns long. The same criterion was used in the [5] study, on the grounds that human annotators could not always agree on whether such small objects represented filopodia or not.

**Figure 7:**
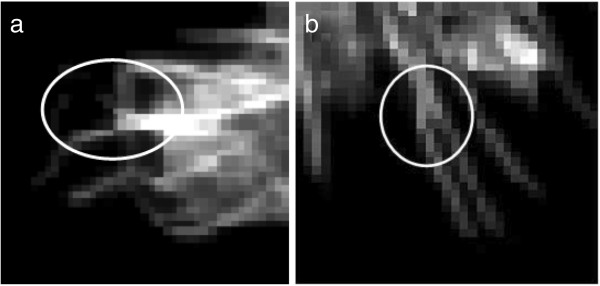
**Two examples of combined filopodia. (a)** cross over along length and **(b)** fused at base.

**Detection of combined filopodia** Combined filopodia represent filopodia that are either fused at the base or that cross over along the length of the filopodia. We call these filopodia “combined” filopodia based on their appearance in the image. They may or may not actually touch in the cell (Figure 7). Detection of these combined filopodia needs additional processing on the split filopodia. The bounding box around each detected filopodium is first obtained. The morphological thinning operation is applied on that bounding box image and the number of endpoints and branch points of the thinned filopodia are calculated. If the number of branch points is greater than 1 and endpoints are greater than or equal to 4, the detected filopodium is considered to be combined.

#### *Step 3: Length estimation of the filopodia*

The split filopodia are morphologically thinned into one pixel connected lines and the lengths of the filopodia are calculated by the area of each thinned filopodium. In this way, combined filopodia are length equivalent to the total length of all filopodia in the combined group. In the Rat2 cells images, the majority of the combined filopodia represent fused or bifurcating filopodia, which share a common base, and are not due to crossing over events. We have considered these fused filopodia as one object and have calculated the length of the fused filopodia using the method detailed above. In the manual count, combined filopodia were also considered as a single object, as they share the same base [5].

## Results and discussion

**Figure 8:**
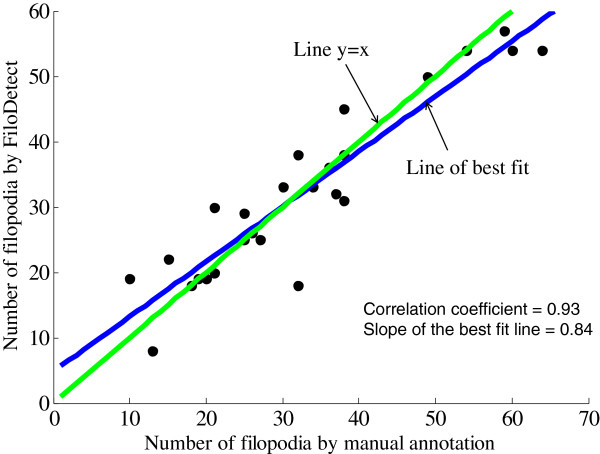
**Scatter plot showing the correlation between manual and automatic count in Rat2 dataset (correlation coefficient = 0.93 and slope of the best fit line = 0.84).** Each point represents one of 26 test images.

**Figure 9:**
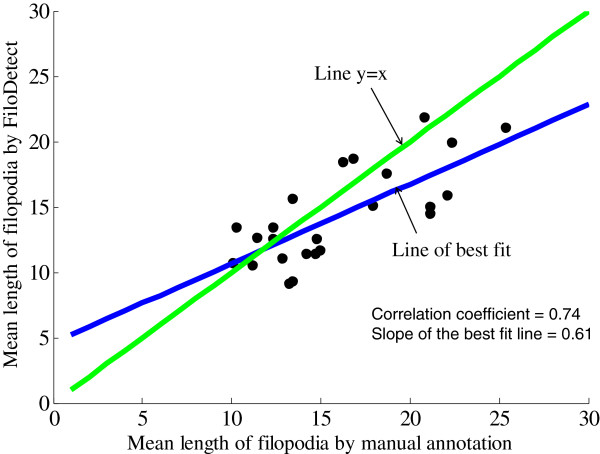
**Scatter plot showing the correlation between manual and automatic length calculation in Rat2 dataset (correlation coefficient = 0.74 and slope of the best fit line = 0.61).** Each point represents one of 25 test images.

**Figure 10:**
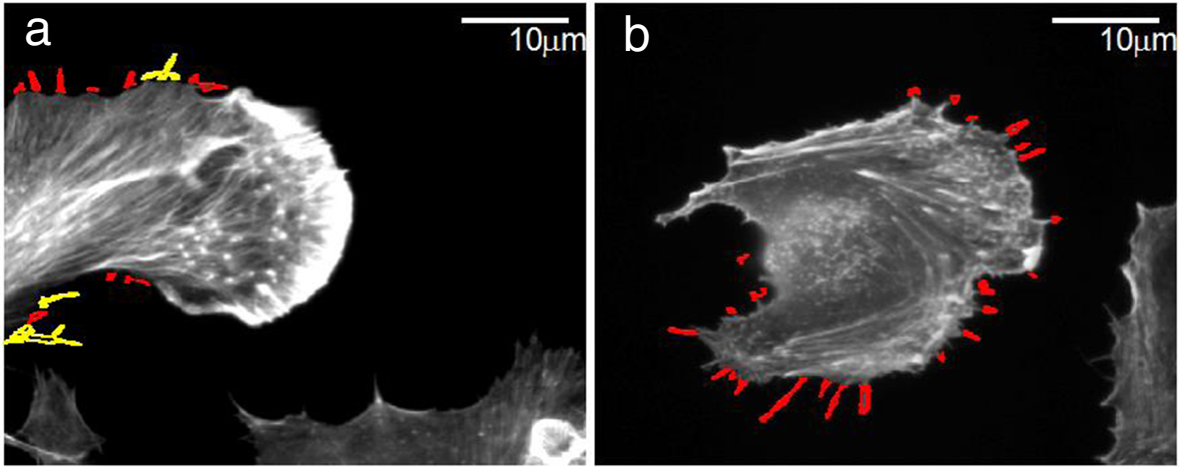
**Example results on images of B16F1 mouse melanoma cells. (a)** detected filopodia of a sample cell of Schober dataset and **(b)** detected filopodia of a sample cell of Svitkina dataset, where single filopodia are shown in red and combined filopodia are shown in yellow.

Filopodia size and number can vary greatly across individual cells. To gauge the ability of the proposed detection system to effectively identify filopodia and substitute for a human expert, we compared the manual count and length measurements of filopodia to the proposed detection method in 26 Rat2 cell images. The density of filopodia of this test set varies from 10 filopodia per cell to 64 filopodia per cell. Also the images are captured in different resolutions. The scatter diagram of Figure 8 shows the automatic and manual filopodia counts on our 26 Rat2 test images. The mean absolute error of FiloDetect counts is 15.69%.^1^ We next compared the manual and FiloDetect-computed lengths of Rat2 filopodia. For the majority of cases, the automatic length measurement generated a slightly lower value than the manual calculation (Figure 9; this plot excludes one cell image which contained very long filopodia, a point agreed upon by both manual annotation and FiloDetect.) This systematic difference in assessed filopodia lengths is due to the fact that there is a certain ambiguity in defining where the base of a filopodium begins on the cell body. The manual annotations appear to consistently begin counting the filopodia pixels further down in the cell body. However, using FiloDetect, the filopodia base points, determined by the size of the structuring element, is the point where the filopodia touches the border of the cell body. Even with this difference between the manual and automatic lengths, the mean lengths obtained across all 26 Rat2 cell test images by automatic versus manual detection, 15.89*μ*m and 19.27*μ*m with standard errors 3.26 and 6.23 respectively, are not statistically different. To further validate the performance of FiloDetect, we applied it to images of B16F1 mouse melanoma cells used in [18] and [19,20]. For the Schober dataset, the *MAE* of the automated count to the manual count is 6.8%, and the mean length obtained by automatic versus manual detection is 13.64*μ*m and 13.89*μ*m with standard errors 2.37 and 2.27 respectively. For the Svitkina dataset, the *MAE* of the automated count to the manual count is 19.96% and the mean length obtained by automatic versus manual detection is 15.95*μ*m and 18.18*μ*m with standard error 1.21 and 2.78 respectively. Figure 10 shows a sample image from each of these datasets and corresponding detected filopodia with FiloDetect. Thus, we can conclude that the automated algorithm designed effectively identifies and measures filopodia length in a manner that replicates results obtained by manual count.

**Figure 11:**
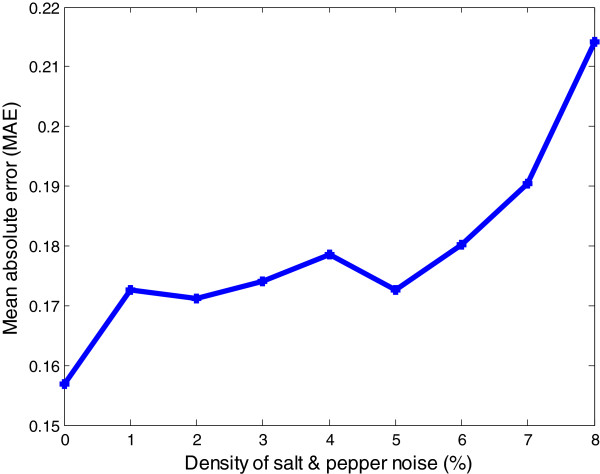
Mean absolute error for different signal to noise ratio.

To detect the robustness of the proposed method we applied FiloDetect on noisy images. We added artificial poisson and salt & pepper noise to our test set. Poisson noise, a common type of noise for confocal microscopy images, is multiplicative noise described by a Poisson distribution, [29,30]. The MAE of FiloDetect count for this noisy test set is 20.23%. To see the performance of FiloDetect system for different signal to noise ratio, we have recorded the MAE on test set with varying degrees of salt and pepper noise. Figure 11 shows the plot of MAE on test set for different percentages of salt and pepper noise.

Next we applied FiloDetect to assess whether increased PI4KIII *β* expression leads to enhanced filopodia number or length in BT549 breast cancer cells. Here we calculated the length and number of single and combined filopodia separately in response to the fact that filopodia are relatively long in BT549 cells, with many filopodia crossing events.

**Figure 12:**
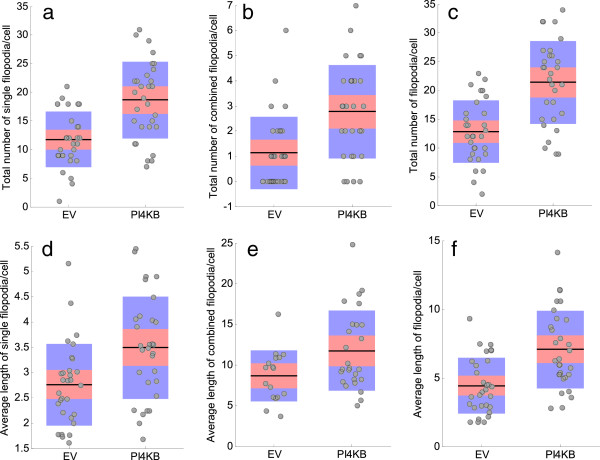
**Boxplots showing different conditions. (a)** total number of single filopodia, **(b)** total number of combined filopodia, **(c)** total number of filopodia, **(d)** average length (in *μ*m) of single filopodia, **(e)** average length (in *μ*m) of combined filopodia and **(f)** average length (in *μ*m) of filopodia between EV and PI4KIII *β*. Points are layed over a 1.96 standard error of mean (95% confidence interval) in pink and a 1 standard deviation in blue.

The box plots in Figure 12(a), (b) and (c) show that the mean number of single, combined and total filopodia per cell are greater in the PI4KIII *β* expressing BT549 cells as compared to the empty vector control cells. From these plots we can see for all cases these results were statistically significant. Using a two-tailed unpaired t-test, the difference in the total number of filopodia per cell in EV and PI4KIII *β* is statistically significant with *p*-value=0.00001. Filopodia length was measured for each group of cells, PI4KIII *β* versus EV, for the single and combined filopodia separately (Figure 12(d) and (e)) and then for all filopodia (Figure 12(f)). The average filpodium length, for all filopodia, was determined to be 4.45 *μ*m for the empty vector controls and 7.10 *μ*m for the PI4KIII *β* expressing cells. When treated separately, the single and crossing filopodia both showed a greater average length in the PI4KIII *β* expressing cells versus the empty vector controls. In all cases these results were statistically significant. By t-test, the difference of average length of filopodia per cell in EV and PI4KIII *β* is statistically significant with *p*-value=0.00001. Therefore, we can conclude from these results that PI4KIII *β* expression leads to a greater number of filopodia, and filopodia that are longer on average, in BT549 breast cancer cells.

## Conclusion

In this paper, we proposed FiloDetect to automate the quantification of filopodia, making more reliable and reproducible the task of quantifying filopodia from static microscopy images. The proposed FiloDetect system was evaluated on Rat2 fibroblast and B16F1 mouse melanoma cell images, manually annotated for filopodia number and length. A comparative analysis of the results shows the good performance of FiloDetect, in both number and length determination. This method was then applied to measure the effect of PI4KIII *β*’s expression on filopodia production in BT549 breast cancer cells. We found that PI4KIII *β* expression leads to an increase in filopodia number and length, suggesting that PI4KIII *β* is involved in driving filopodia production in the cell. When overexpressed, PI4KIII *β* may promote cancer cell metastasis, as filopodia are a characteristic of invasive cells.

Although FiloDetect compared favorably to manual annotations and was accurate enough to carry out the PI4KIII *β* analysis, further improvements may be possible. In Costantino’s work on detecting filopodia on neural growth cones [10] they found that segmentation based on edge detection was superior to intensity based thresholding–although both are options in their software. In pilot studies, we did not find an advantage to edge detection. However this might be true for other image sets. Adaptive intensity thresholding methods, where the threshold varies for different parts of the image, or methods that combine intensity and edge information might also yield improvements. Because the filopodia are comparatively small objects in typical images, and because it can be difficult for morphological analysis to correct for errors in segmentation, high quality segmentation is key to our approach. A completely different approach would be to forego segmentation and use a tracing-based approach to delineate filopodia. In the neurite detection literature, tracing-based approaches are generally considered to be the most accurate, although their computational burden is higher than that of morphology-based approaches.

Another area for possible improvement is in the untangling of combined filopodia. Following the policy of our previous manual annotations, we have not attempted untangling. However, some combined filopodia are truly physically joined, whereas others are really separate but overlap visually. By analyzing joined structure in more detail, it may be possible to discriminate between these cases. We have conducted preliminary analysis of 3D image stacks, to see if they might be informative in this regard. However, segmenting the cell is much more difficult in this case, because each layer of the stack contains differing and only partial information on where the cell boundaries are.

Filopodia are just one of many cytoskeletal features that are biologically relevant and that we might want to quantify automatically from images. For instance, it would be of interest in the study of cytoskeleton remodelling to be able to automatically define and measure the relative size/cellular proportion of a cell’s lamellipodium, which defines the flat and broad cellular protrusion containing a meshwork of branched F-actin found at the leading edge [2]. In addition, it would be useful to develop an algorithm that is able to quantify the number/proportion of stress fibers, contractile acto-myosin structures, which span the length of a cell, and are involved in adhesion and motility [31]. Robust and automated quantification of the size of the lamellipodium and the number of stress fibers in a cell under genetic and chemical perturbations, along with the measure of filopodial protrusions would allow a broader study of events of cytoskeletal rearrangement. Also, it would be interesting to see if our algorithm to measure filopodia number and length could be applied in a live cell imaging context, allowing real-time actin dynamic remodelling events to be studied quantitatively.

## Availability and requirements

Algorithms were implemented in Matlab2009. The FiloDetect system and some sample cell images are available at http://www.perkinslab.ca/Software.html. There is no restrictions on non-commercial use of this software.

## Endnote

^1^ Here we define, MAE $=\sum_{i=1}^{N}|M_{i}-F_{i}|/NM_{i}$ where *M*_*i*_ = *M*_1_,*M*_2_,⋯*M*_*N*_ are the manual counts and *F*_*i*_ = *F*_1_,*F*_2_,⋯*F*_*N*_ are the FiloDetect counts for *N* different cells.

## Competing interests

The authors declare that they have no competing interests.

## Authors’ contributions

SN and TJP proposed and designed the filoDetect method and drafted the manuscript. AM generated and imaged the Rat2 and BT549 cell lines used in this study and helped to draft the manuscript and to generate manual annotation. JML helped to generate manual annotation and to draft the manuscript. All authors read and approved the final manuscript.
